# Color correction methods for underwater image enhancement: A systematic literature review

**DOI:** 10.1371/journal.pone.0317306

**Published:** 2025-03-10

**Authors:** Yong Lin Lai, Tan Fong Ang, Uzair Aslam Bhatti, Chin Soon Ku, Qi Han, Lip Yee Por

**Affiliations:** 1 Department of Computer System and Technology, Faculty of Computer Science and Information Technology, Universiti Malaya, Kuala Lumpur, Wilayar Persekutuan, Malaysia; 2 School of information and Communication Engineering, Hainan University, Haikou, Hainan, China P.R.; 3 Department of Computer Science, Universiti Tunku Abdul Rahman, Kampar, Perak, Malaysia; 4 School of Computer Science, Shenyang Aerospace University, Shenbei, Shenyang, China P.R.; Whale Wave Technology Inc, CHINA

## Abstract

Underwater vision is essential in numerous applications, such as marine resource surveying, autonomous navigation, objective detection, and target monitoring. However, raw underwater images often suffer from significant color deviations due to light attenuation, presenting challenges for practical use. This systematic literature review examines the latest advancements in color correction methods for underwater image enhancement. The core objectives of the review are to identify and critically analyze existing approaches, highlighting their strengths, limitations, and areas for future research. A comprehensive search across eight scholarly databases resulted in the identification of 67 relevant studies published between 2010 and 2024. These studies introduce 13 distinct methods for enhancing underwater images, which can be categorized into three groups: physical models, non-physical models, and deep learning-based methods. Physical model-based methods aim to reverse the effects of underwater image degradation by simulating the physical processes of light attenuation and scattering. In contrast, non-physical model-based methods focus on manipulating pixel values without modeling these underlying degradation processes. Deep learning-based methods, by leveraging data-driven approaches, aim to learn mappings between degraded and enhanced images through large datasets. However, challenges persist across all categories, including algorithmic limitations, data dependency, computational complexity, and performance variability across diverse underwater environments. This review consolidates the current knowledge, providing a taxonomy of methods while identifying critical research gaps. It emphasizes the need to improve adaptability across diverse underwater conditions and reduce computational complexity for real-time applications. The review findings serve as a guide for future research to overcome these challenges and advance the field of underwater image enhancement.

## Introduction

As the demand for marine resources rises, underwater vision finds extensive applications in fields such as marine resource surveying, underwater autonomous navigation, underwater object detection, and underwater target monitoring. However, raw underwater images often encounter quality degradation such as color deviations, low contrast, and blurring [1–5], significantly impacting subsequent processing and information extraction.

According to the underwater imaging model developed by Jaffe-McGalmey, the light captured by the camera comprises three components: the light directly reflected from the target object, the forward scattered light deviated at a small angle from the target object, and the backscattering light originating from ambient light that interacts with underwater suspended particles [6]. Underwater image degradation issues are mainly attributed to the absorption and scattering effects of light during the propagation of the underwater medium. Water absorbs different wavelengths of light to varying degrees, resulting in severe color deviations in underwater images [1,2]. Red-colored light with the longest wavelength diminishes the quickest, disappearing completely at approximately 4 – 5 m [3]. In contrast, green and blue light travel further, causing a blue-green color bias in underwater images. This uneven light attenuation with depth results in color deviations and loss of valuable visual information.

Additionally, organic matter and suspended particles in water cause significant light scattering, which blurs and fogs underwater images [4]. Unlike images captured in air, underwater images face greater variability due to the complex optical properties of water. Despite advances in imaging hardware technologies, addressing the challenges of color bias and scattering in underwater environments remains a significant problem [5]. Enhancing underwater images, especially by correcting color deviations, is critical for improving their quality for practical applications.

The core research questions addressed in this systematic literature review (SLR) involve identifying existing methods used in underwater image enhancement for color correction and analyzing their shortcomings. Understanding the current state of knowledge and challenges in underwater image enhancement is crucial for grasping how to address color deviations effectively. This subsequently leads to the research objectives of exploring and investigating the existing landscape of methods used in underwater image enhancement for color correction and examining their shortcomings.

To conduct a systematic literature review, we employed a systematic approach to examine a range of scholarly databases using a specific search string. This SLR aims to present an in-depth analysis of existing methods for underwater image enhancement for color correction, identifying gaps, trends, and emerging directions within the realm of underwater image enhancement.

The key contributions of this SLR are as follows:

- This paper presents a comprehensive and organized overview of methods used for underwater image color correction, classifying them into physical model-based, non-physical model-based, and deep learning-based approaches. By offering a structured taxonomy, it aids researchers, practitioners, and developers in understanding the practical applications of these techniques for addressing color deviations in underwater images.
- The review provides a detailed analysis of the limitations inherent to each method, focusing on key challenges such as data dependency, algorithmic constraints, computational demands, and inconsistent performance. By highlighting these issues, the paper offers a critical assessment of the current state of underwater image enhancement research and identifies areas that warrant further exploration and innovation. This gap identification serves as a foundation for guiding future studies aimed at advancing the field.
- This paper highlights the growing prevalence and potential of deep learning-based techniques, reflecting advancements in image processing. By comparing these data-driven methods with traditional techniques, the review provides valuable insights into the future direction of research in underwater image enhancement. This emphasis on emerging trends helps set the stage for further innovations and developments in the field, ensuring that future research builds on the most promising and effective techniques identified in the review.

The paper’s structure is arranged with four main sections: methods, results, study taxonomy, and finally the conclusion.

## Methods

The methodology used to conduct this SLR is based on the Preferred Reporting Items for Systematic Reviews and Meta-Analyses (PRISMA) framework [7], as detailed in the PRISMA 2020 checklist in S1 Table. Employing the PRISMA approach ensured a transparent and structured procedure for article selection, provided with step-by-step guidelines from identification to screening to inclusion. This is expected to provide more transparent, comprehensive, and consistent reporting of systematic reviews. The methodology was designed to thoroughly explore the methods used in underwater image enhancement for color corrections from existing literature, consisting of nine main stages as discussed in the following sub-sections.

### Define research questions and objectives

The core objectives of this SLR are to identify methods used in underwater image enhancement to tackle color deviation issues and their shortcomings. The research questions and research objectives are as follows:

Research Questions:

1. What are the existing color correction methods used for enhancing underwater images?
2. What are the shortcomings of the identified methods for enhancing underwater images?

Research Objectives:

1. To review the existing color correction methods used for enhancing underwater images.
2. To examine the shortcomings of the identified methods for enhancing underwater images.

### Identify information sources/databases

A comprehensive search was carried out using a diverse range of databases, including Academic Search Elite @EBSCOhost, Education Research Complete @EBSCOhost, Web of Science, IEEE Xplore, Scopus, Science Direct, MDPI, and SAGE Journals. These databases were used to obtain relevant journal articles and conference papers for this SLR.

### Develop the search strategy/search terms

A search strategy was developed based on the research question and objective using a combination of keywords and controlled vocabulary terms related to underwater image and enhancement methods. Secondary keywords to enhancement, such as non-physical, fusion, deep learning, algorithm, and model, were used to widen the search. Boolean operators, including ‘AND’ and ‘OR’, were used to merge and refine the search terms. The chosen search string for this study is “underwater image” AND (“enhancement” OR “non-physical” OR “fusion” OR “deep learning” OR “algorithm” OR “model”).

### Define inclusion and exclusion criteria

Screening criteria were established to decide which studies would be included in or excluded from the review. Table 1 outlines the screening criteria. The identified databases were used for the search on March 29, 2024. The decision to include only articles from the most recent 15 years, spanning from 2010 to 2024, is based on several considerations. This period represents a pivotal phase in the evolution of underwater image color correction techniques, characterized by significant advancements in algorithmic approaches and computational capabilities. Limiting the review to this 15-year range ensures a balance between including a broad selection of relevant studies and avoiding information overload. This approach enables the review to remain focused and manageable while capturing both foundational research and recent innovations, providing a comprehensive understanding of the advancements and trends in the field.

**Table 1:** Screening criteria applied for the study selection process.

Inclusion Criteria	Exclusion Criteria
Studies were published between 2010 and 2024.	Studies primarily focus on hardware-based underwater image enhancement methods, tools, or techniques.
Studies published in the English language.	Studies that do not propose any methods but use and compare the existing methods.
Peer-reviewed journal or conference papers.	Studies that do not have an evaluation carried out for their proposed methods.

Additionally, only peer-reviewed journal or conference papers were included in this SLR to ensure the credibility and reliability of the sources, as these publications undergo rigorous evaluation by experts in the field. This helps to minimize the risk of using inaccurate or biased information.

### Screening and selection process

The selection process involved four main stages: identification, screening, eligibility, and inclusion. The selection process was first conducted by one reviewer, followed by a second reviewer verifying a random sample of screened records to ensure that the screened studies are consistent.

Firstly, the identification process involved using the search string in the title, abstract, and keyword filters to obtain relevant studies from the identified databases. A Microsoft Excel document was used to record the titles, abstracts, DOIs, or URLs of the retrieved studies. It then proceeded to the screening stage, where the studies were evaluated for their relevance to the research question and objective based on their titles and abstracts. Studies that were duplicated or irrelevant were manually excluded from further consideration.

In the eligibility stage, the remaining studies underwent a full-text review to assess their adherence to the inclusion and exclusion criteria. The final inclusion stage compiled all shortlisted studies deemed suitable for the review.

### Quality assessment

A quality assessment of the selected studies was conducted to evaluate their strengths and weaknesses, focusing on methodological conduct and analysis. Quantitative studies were assessed for quality and potential bias using the Mixed Methods Appraisal Tool (MMAT) [8] was used. For qualitative studies, the Critical Appraisal Skills Programme (CASP) checklist [9] was applied to assess trustworthiness, relevance, and contextual value. Both MMAT and CASP checklists involved five criteria to ensure a comprehensive and robust quality evaluation on the selected studies. This step is to strengthen the depth and integrity of this review.

### Data extraction

The review team extracted data that are relevant to the research questions from the selected studies, focusing on methods for color correction in underwater image enhancement. The titles of the studies and the specific methods used to address color deviations were extracted and identified. This provides a preliminary understanding of the techniques employed in the selected studies.

### Data synthesis and analysis

The data synthesis process involved summarizing and evaluating the findings from the review of selected studies, presented in table format. The content analysis technique was used to identify common themes among the identified methods.

It is highlighted that this SLR aims to provide a detailed discussion of the color correction methods used for underwater image enhancement. Specific statistical techniques, such as evaluation of the effect and meta-regression, were not covered within the scope of this review.

### Identify research gaps and contributions

This SLR revealed the current state of knowledge on the methods used in underwater image enhancement for color correction, offering a comprehensive view to guide future research in identifying areas where further investigation is needed. By analyzing the methods used for color correction in the literature, the gaps and limitations in existing strategies can be identified. These research gaps will serve as a foundation for further investigations, supporting the design, development, and improvement of more robust and effective color correction methods in underwater image enhancement, thereby enhancing the practical applications of underwater vision.

## Results

### Study selection

**Fig 1:**
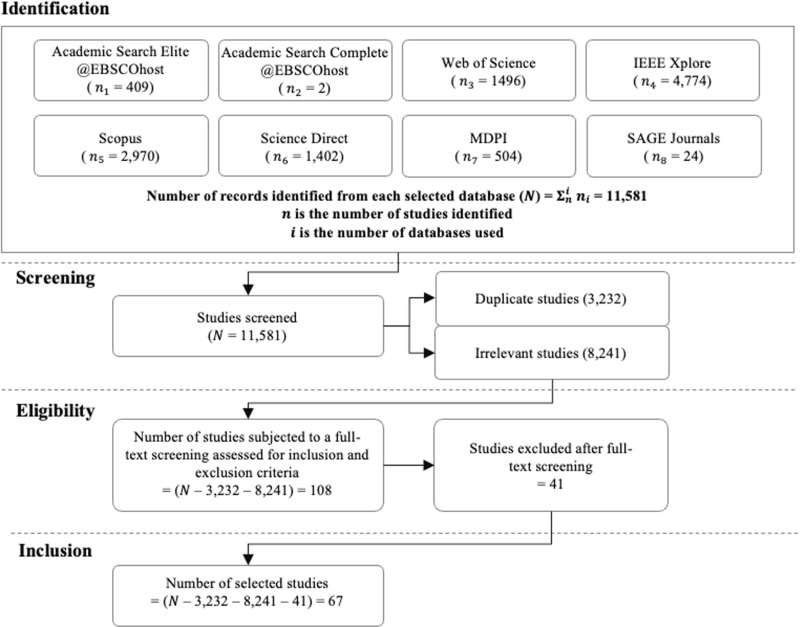
PRISMA flowchart for the study selection process.

Fig 1 presents the PRISMA flowchart, which outlines the four stages involved in this SLR: identification, screening, eligibility, and inclusion. During the initial identification stage, a total of 11,581 studies were obtained from the database search using the chosen search string of “underwater image” AND (“enhancement” OR “non-physical” OR “fusion” OR “deep learning” OR “algorithm” OR “model”).

Following the identification stage, a total of 3,232 duplicate studies and 8,241 irrelevant studies were excluded from the review (S3 Table). A full-text review was conducted for the remaining 108 studies to assess their eligibility based on the inclusion and exclusion criteria, which subsequently excluded another 41 studies. Finally, the inclusion stage included the remaining 67 studies that met all the inclusion and exclusion criteria in this SLR (S4 Table).

**Fig 2:**
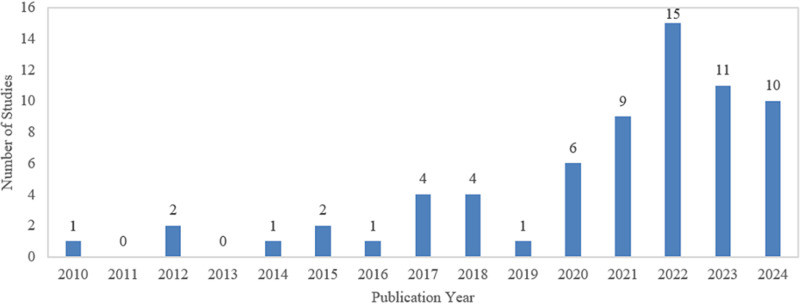
Distribution of selected studies by publication year.

Fig 2 illustrates the distribution of selected studies by publication year between 2010 and 2024. It shows a steady increase in the number of studies over the past year. From 2016 onwards, there is a noticeable upward trend, peaking in 2022 with 15 studies. This indicates growing interest in and advancements in the field of underwater image enhancement in recent years.

**Fig 3:**
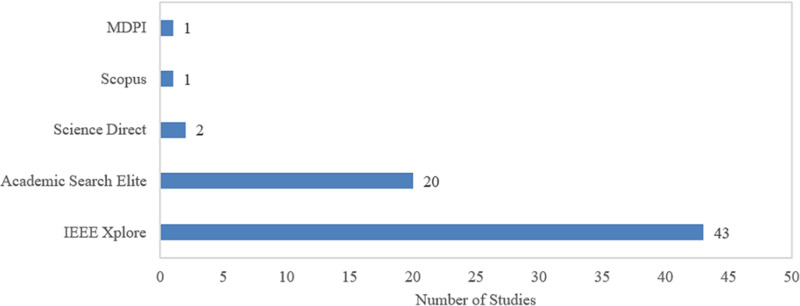
Distribution of selected studies by database.

Fig 3 depicts the distribution of selected studies by database. Out of the 67 selected studies, IEEE Xplore emerges as the leading source, contributing 43 studies, followed by Academic Search Elite with 20 studies. Other publishers like Science Direct, Scopus, and MDPI contribute one to two studies, respectively. This distribution highlights the dominance of IEEE Xplore and Academic Search Elite in publishing research related to underwater image enhancement, reflecting their central role in disseminating knowledge within this domain.

### Quality of included studies

The quality assessment of the selected studies in this review is provided in the Assessment of Study Quality (S2 Table). Among the 57 quantitative studies, 41 studies (72%) fulfilled all the criteria outlined by the MMAT tool. The remaining 16 studies (28%) were of medium quality, with MMAT scores ranging between 60% and 80%, indicating partial satisfaction with the criteria. This is mainly due to the inability to meet criterion 1.2 (sample representativeness), where some studies did not explicitly state or discuss the datasets used in their experiments. This may raise questions about whether the datasets used represent a valid sample to justify the effect of their proposed method in tackling color deviations in underwater images. However, no quantitative studies were evaluated as being of low quality.

For the qualitative studies, 2 studies (20%) achieved high quality, fulfilling all the criteria outlined by the CASP tool. The remaining 8 qualitative studies (80%) were of medium quality due to a lack of description of the data collection and analysis processes. These studies mainly focus on the conceptual design and analysis of the proposed methods for correcting color deviation and enhancing underwater images.

### Research question 1: What are the existing color correction methods used for enhancing underwater images?

A total of 13 color correction techniques were reported in the articles, each offering a distinct approach to compensating for light attenuation and color deviations in underwater images. Table 2 lists these methods along with the related articles.

**Table 2:** Color correction methods used for enhancing underwater images and related articles.

Underwater Image Color Correction Method	Related Articles
Underwater optical imaging properties-based	[10–17]
Color constancy-based	[18–24]
Red channel compensation-based	[20,25–27]
Adaptive color correction-based	[28–38]
Histogram equalization-based	[39–47]
Color transfer-based	[48–51]
Multi-color spaces-based	[46,52–55]
Color opponent compensation-based	[56,57]
Fusion-based	[18,20,22,25,29,38,45,50,55,58]
Convolutional neural network (CNN)-based	[43,46,49,52,53,59–66]
Generative adversarial network (GAN)-based	[57,67–74]
Transformer-based	[75,76]
Deep Q-learning (DQL)-based	[77]

The underwater optical imaging properties-based methods primarily rely on compensating for depth or wavelength variations using a physical model that factors in underwater optical properties, including scene depth and background light. These properties are used to build a physical model to reconstruct the underwater images accurately. Depth estimation plays a critical role in these methods, as the varying attenuation rates of different color channels (red, green, and blue) are depth dependent. The red channel attenuates most rapidly, followed by the green and blue channels. Therefore, accurately estimating the scene depth for each pixel is crucial to correctly adjusting the color balance. For example, methods introduced in [11–15] use depth estimation to obtain specific attenuation coefficients at different depths to rectify color deviations effectively. The effectiveness of these methods is highly dependent on the accuracy of depth and background light estimations. Additionally, some methods compensate for local surface reflectance statistics to restore the actual reflectance of objects in underwater images based on the physical model [10]. Other methods leverage multiple images captured at different exposure times. This approach collects spectral information across all color channels to enable more effective adjustments for color attenuation [16,17].

The color constancy-based methods operate on the assumption that the illumination in a scene is known or can be estimated, allowing for color correction based on the characteristics of the light source. Several algorithms have been developed in this category, including underwater white balance (UWB), Gray World, and Retinex. UWB adjusts the RGB channels of an image to balance their respective intensities such that white objects appear white after correction. UWB approaches can be based on differences in mean RGB values [18–20] or standard deviation ratios [21] to counteract color biases. For Gray World assumption, it assumes that the average reflectance of all objects in the scene is achromatic, thus ensuring that the average intensity of each color channel is roughly equal. For underwater scenes with rich colors, this method performs well; however, in less colorful scenes, local entropy-constrained Gray World approach can be employed to assess the local color richness based on the local entropy value and apply the Gray World algorithm locally only for regions with high color richness to avoid overcompensation of the red channel [22]. Inspired by human vision, Retinex theory divides images into reflectance and illumination components for detail enhancement and color correction separately [23]. While Retinex-based methods are effective in addressing color imbalances, they may introduce artifacts in complex underwater environments due to their neglect of wavelength-dependent light attenuation [24].

Red channel compensation-based methods aim to restore the red component by using information from the other color channels, such as blue and green, which are less attenuated in underwater environments. This is given the prior assumption that the red channel is most affected by light attenuation in underwater environments due to its long wavelength [3]. One method, as described in [25], compensates for the red channel by identifying the dominant channel, either blue or green, and adjusting the red channel based on the attenuation ratio. Another approach determines the similarity between the red and the dominant channel to calculate the compensation level required for each pixel [26,27]. Since the green channel is generally less affected by attenuation, it is often used to infer missing red information [20]. To avoid overcompensation, color constancy algorithms, such as Gray World assumption, are subsequently applied to correct any residual color imbalances.

Adaptive color compensation-based methods, in contrast, do not rely on predefined assumptions about light attenuation. Instead, they independently adjust each color channel, making them particularly useful in complex underwater scenes where attenuation levels vary. These methods employ a variety of techniques, such as attenuation matrices [33], gain-based compensation [29–31], and adjustments based on global or local channel difference values [32]. For example, in blue-dominant underwater images, the least attenuated blue channel is used to guide corrections for the red and green channels [34,35]. Moreover, the types of color deviation in underwater images, such as greenish, bluish, yellowish, or undistorted, can be assessed using methods like color cast factor (CCF) [35] or by identifying the best background area in the raw underwater image using the Quadtree search method [36]. Other methods for adaptive color correction include using an attenuation map estimated by comparing the intensity of the actual underwater scenes and that captured by the camera to analyze the light attenuation level [37] and applying a color dominancy reducing factor to the most attenuated color channel to mitigate color dominance globally [38].

Histogram equalization-based methods are widely used for improving contrast and color balance of images. They work by adjusting the intensity histograms of each RGB component towards a more uniform distribution, which helps balancing the overall color and improve image visibility. Typically, the histogram of the inferior color channel (red) is deviated towards the lower intensity scale, while the histogram of the dominant color channel (green or blue) tends to concentrate at the higher end of the intensity scale. In one approach described in [39,40], histogram stretching is implemented across the full range of the underwater image, from 0.2% to 99.8% of the image histogram, with the inferior color channel stretched towards the upper end and the dominant color channel shifted towards the lower end. However, this method may produce images with darker intensity and potential over-enhancement in specific areas. To mitigate under- and over-enhancement effects, the stretching process follows the Rayleigh distribution and constrains the output intensity levels to 5% of the minimum and maximum limits [41]. Recent works extend the classical histogram equalization framework, such as utilizing sub-interval linear transformation to suppress shadows and highlights [42], introducing a compression operation with a simple log-operation to mitigate over-enhanced artifacts [43], implementing recursive adaptive histogram modification (RAHIM) to divide each RGB component into eight columns and process them with overlapping histograms according to the Rayleigh distribution [44], and employing a dual-interval histogram approach to enhance foreground and background sub-images separately before integration [45].

The color transfer-based methods adjust the color of an image by transferring the mean and standard deviation of its colors from a reference image with similar visual characteristics [48–51]. The choice of a visually similar reference image is critical to achieving satisfactory enhancement results. Recent developments in this field have introduced regularization constraints, enabling localized color adjustments rather than relying solely on global statistics [48]. Furthermore, deep learning algorithms such as the latent consistency learning network (LCL-Net) have been proposed to automate reference image selection and improve color transfer accuracy by ensuring consistency between the reference image pool and the raw underwater images [49].

Many underwater image enhancement methods focus on processing features in the RGB color space to effectively mitigate scattering and color deviation issues [52,53]. However, working solely in RGB space may not be able to capture all critical parameters required for accurate color correction, limiting the effectiveness of the enhancement techniques. The multi-color spaces-based methods extract features from multiple color spaces, such as RGB, HSV (Hue, Saturation, and Value), and Lab color spaces, each providing distinct information [46,52–55]. Specifically, the RGB color space divides colors into red, blue, and green channels; the HSV color space allows for an intuitive representation of hue, saturation, and value (or brightness) of the image; and the Lab color space offers an improved and diverse color representation by expressing colors based on lightness (L*) and color-opponent channels (a * and b*), whereby a * indicates red-green variation and b * indicates blue-yellow variation. By combining features from multiple color spaces, these methods enhance the visual quality and color accuracy of underwater images, which are usually more aligned with human visual perception.

The color opponent compensation-based methods are based on the concept that opposing color channels, such as green-red (G-R), yellow-blue (Y-B), cyan-red (C-R), and black-white (B-W), work together to balance each other out. Color opponent compensation-based methods exploit this relationship to correct color imbalances in underwater images. To counteract color channel loss due to water absorption, these methods adjust the opponent color channels such as green-red (G-R) towards a zero local mean [56,57]. This can be achieved using Lab [56] and LMS (long, medium, and short) color spaces [57]. For instance, in the Lab color space, if an underwater image appears greenish due to a loss of red channel, the method compensates by adjusting the a * channel back towards zero, effectively recovering lost red information.

The fusion-based methods enhance color correction by combining multiple processed versions of a single raw image. The idea is to create different input images that emphasize various aspects such as contrast, saturation, detail, and luminance, and then fuse them to produce a more realistic and visually pleasing output. The approach involves generating multiple inputs and weights from the raw underwater image and applying pixel-level fusion techniques such as the Laplacian pyramid and Gaussian pyramid to produce the final image. As described in various works [18,25,29,38], three processed versions of an image can be generated from a single raw input: a locally contrast-enhanced version, a globally contrast-enhanced version, and a sharpened version. Four fusion weights, representing global contrast, local contrast, saturation, and saliency, are then computed and normalized. These weight maps are used in multiscale fusion to merge the input images, producing an output with more realistic colors and improved contrast. Another approach generates two inputs from a single image using two different color correction methods [50,58]. For example, one input undergoes minimal color correction, while the other receives maximal correction [50]. These are then fused to complement each other for optimized color enhancement. In addition, images in different color spaces like RGB, HSV, and Lab can be combined to complement each other to correct saturation, intensity, and luminance [55].

The convolutional neural network (CNN)-based methods are known for their feature extraction capabilities. CNNs are deep learning architectures that could learn underwater image statistics and map raw underwater images to their enhanced counterparts for image enhancement. For instance, a two-branch network has been designed to extract distinct features from the same input image through separate branches [43]. Specifically, one branch corrects global color deviation while the other enhances local contrast. These branches are then combined using global residual-guide biases. Similarly, the two-stage underwater image enhancement CNN based on structural decomposition (UWCNN-SD) improves the estimation of transmission maps and background light, allowing for adaptive color correction across various underwater scenarios [59]. CNN-based methods also leverage parallel multi-color space encoders, using color spaces such as RGB, HSV, and Lab to maximize the information utilized for color correction [46,52,53]. A multi-path residual block enables RGB enhancement while establishing inter-color channel connections via attention-based cross-links, facilitating more effective color correction [60]. Another model, the grouped color compensation encoder and channel fusion decoder (GCCP) uses group convolutional and a color compensation layer to correct color deviations adaptively while maintaining a lightweight structure [61]. CNN models also often incorporate multiscale techniques to merge features using attention-based fusion modules [62,63]. Some CNN-based methods are designed to enhance generalization in complex underwater scenes without requiring extensive training data. For example, the fast adaptive self-supervised color mapping map (CLM-Net) can learn domain-specific degradation parameters from just ten images [64], while the latent consistency learning network (LCL-Net) selects optimal color transfer templates based on latent mutual consistency [49].

While CNN-based methods heavily count on the availability of training data, generative adversarial network (GAN)-based methods tackle underwater color deviation through two main strategies: synthesizing training images and translating images between underwater and in-air domains. GANs use a generator and a discriminator to adjust weight parameters, producing enhanced images with color correction and preserved detail. WaterGAN generates synthetic underwater image pairs from in-air images and depth maps to train a color correction network [67]. A depth-conditioned GAN expands on this by generating corresponding depth maps along with underwater images as input to the generator, addressing the problem of incomplete green hue recovery [68]. Another GAN-based method incorporates the statistical background color distribution of underwater images in addition to the in-air images and depth maps to produce synthetic underwater images [69]. CycleGAN-inspired models transfer underwater scene color to in-air scenes without requiring paired underwater images for training, using a weakly supervised color transfer methods [70]. An adaptive degradation module further improves CycleGAN by allowing it to learn various degradation patterns from unpaired underwater images, refining image-to-image translation for better color correction [71]. A deep transfer learning model based on CycleGAN also converts underwater images to the in-air domain and applies a restoration model for color correction [72]. Multiscale generators and dual discriminators are commonly used in GAN-based methods to eliminate color deviation at both the global and local levels without over-enhancing the image [73,74].

The transformer-based methods, which rely on self-attention mechanisms, excel in modeling global interactions within underwater images. Unlike CNNs that focus more on local features, these methods are particularly effective for long-range dependency modeling and emphasizes on global feature extraction [75,76]. The U-shape transformer model, proposed in [76], integrates channel-wise multi-scale feature fusion and spatial-wise global feature modeling, allowing it to better handle severely attenuated color channels and spatial areas for improved color correction. To address the limitations of transformers in local context extraction, reinforced feed-forward networks (R-FNN) are incorporated into the reinforced LeWin Transformer block (RLTB)-based encoder and decoder structure, improving both global and local feature capabilities.

The deep Q-learning (DQL)-based methods, a type of reinforcement learning, integrates Q-learning with deep neural networks to determine the most favorable sequence of color correction actions for underwater images. In [77], the task is formulated as a Markov Decision Process (MDP), allowing the DQL approach to automatically select appropriate global color correction actions. Trained with paired underwater images, the network learns the optimal sequence of actions for color correction without explicit supervision for intermediate steps. The actions include conventional image processing operations and white balance techniques, incorporating the predefined adjustment settings such as six basic adjustments for brightness, contrast, and color saturation, along with six settings for white balance to either increase or decrease two color components. This approach addresses the generalization limitations of deep learning-based approaches in diverse underwater scenes, offering robust performance even in presence of unknown color distributions.

### Research question 2: What are the shortcomings of the identified methods for enhancing underwater images?

Based on the methods identified in response to Research Question 1, the shortcomings of each method are discussed as follows:

#### Underwater optical imaging properties-based.

Underwater optical imaging properties-based methods rely on accurate modeling of light interactions in water, such as absorption and scattering coefficients. These coefficients are not only difficult to measure but also highly variable depending on location, depth, time of day, and even the specific imaging device used [11]. This variability makes it difficult to achieve consistent results across different environments. Although these methods aim to correct color deviations, they often fail to completely remove color artifacts in complex underwater scenes [15]. Simulating light propagation is also computational intensive, limiting real-time applications. Specifically, in highly turbid water, light scattering and attenuation further reduce image quality [14].

#### Color constancy-based.

Color constancy-based methods aim to keep colors consistent under different lighting conditions. However, they often fail to account for wavelength-dependent light absorption. This is important because underwater environments absorb certain color channels more quickly than others. In scenes without neutral colors, color constancy-based methods tend to result in inaccurate color correction and unwanted artifacts [24]. For example, using a white balance algorithm without considering the strong attenuation of the red channel can result in over-enhancement [25]. Moreover, these methods typically assume uniform lighting conditions, which is rare in underwater settings where illumination is highly uneven. As a result, they often struggle with pronounced color casts and uneven lighting conditions, leading to suboptimal color reproduction.

#### Red channel compensation-based.

Red channel compensation-based methods focus on addressing the severe attenuation of the red channel in underwater images. However, simplifying the color correction problem to solely focus on the red channel can be overly simplistic. These methods assume that the red channel is always the most attenuated, whereby this assumption might not hold true in all underwater conditions, especially in shallow or less turbid waters. This simplification in the approach can cause the red channel to be over-enhanced, leading to unrealistic color correction. Furthermore, in low-light conditions, where the red channel is almost completed absent, these methods are less effective [26].

#### Adaptive color correction-based.

Adaptive color correction-based methods adjust colors based on predefined settings such as attenuation matrices [33], gain-based compensation [29–31], and global or local channel difference values [32]. These methods are designed to adapt to different underwater conditions, offering some degree of flexibility. However, the effectiveness of these methods is often limited by oversimplifications. They typically fail to account for factors such as non-linear light absorption and scattering. As a result, directly adjusting colors based on fixed parameters without considering the image degradation mechanisms can cause unnatural color corrections and over-enhancement [29]. They also struggle in low-light or very turbid waters, where much of the color information is missing or severely degraded [33,36].

#### Histogram equalization-based.

Histogram equalization-based methods aim to enhance image contrast by redistributing pixel intensities. However, while they can effectively improve contrast in some regions, they often introduce noise and artifacts, especially in low-light areas of underwater images. Over-enhancing contrast may result in a loss of detail in some regions. For example, stretching the histogram without considering the overall color balance can cause certain regions in the images to appear unnaturally dark or over-enhanced [39,40]. In addition, these methods are less effective when handling underwater images with significant noise, often leading to suboptimal enhancement results [42].

#### Color transfer-based.

Color transfer-based methods rely on a reference image to guide the color correction of the target image. However, the effectiveness of these methods heavily depends on the quality and similarly between the reference image and target image in terms of color distribution and lighting conditions. If there is a significant mismatch, the transfer can produce unrealistic color mappings and poor visual outcomes [49]. In addition, these methods also tend to be computationally intensive, particularly when applied to large datasets or high-resolution images, making them impractical for real-time applications [48]. The need of fine-tuning to match varying underwater conditions further amplifies the computational cost.

#### Multi-color spaces-based.

Multi-color spaces-based methods use different color spaces to enhance underwater images. However, selecting and combining multiple color spaces is challenging, especially in the context of underwater environments with highly variable lighting conditions. Managing various color spaces adds complexity, increasing the difficulty of achieving optimal results, particularly in low-light or turbid underwater conditions [53–55]. The information from various color spaces may still be insufficient to recover the degraded underwater images effectively. As a result, these methods often produce inconsistent results across different underwater environments. In addition, compared to simpler, single-color space methods, the added computational burden of processing data from multiple color spaces can be a significant drawback.

#### Color opponent compensation-based.

Color opponent compensation-based methods mimic human visual system by balancing opposing color channels like green-red (G-R), yellow-blue (Y-B), cyan-red (C-R), and black-white (B-W) [56,57]. While this approach can correct moderate color deviations, they assume all individuals perceives color similarly, which may not always hold true. In the case of severe underwater color deviations, balancing opposing channels may be enough to achieve optimal results. Specifically, in deep or highly turbid water, light scattering and attenuation further reduce image quality.

#### Fusion-based.

Fusion-based methods enhance underwater images by merging multiple inputs from a single raw image. Although they can produce good visual results, they are computationally demanding and time-consuming, making them less practical for real-time applications. Accurate computation of fusion weights is essential for obtaining optimal results [18,25,29,38]. However, errors in this process can introduce artifacts like unnatural colors or distorted features. Managing different color spaces during fusion also adds more complexity, often leading to inconsistent color correction [55]. Fusion methods may also struggle in highly turbid water or low-light conditions, leading to variable performance in different underwater environments.

#### CNN-based.

CNN-based methods learn complex patterns from data to enhance underwater images. However, these methods require large amount of labeled training data, which is often difficult to obtain for underwater scenes due to varying water conditions and lighting [43,49]. In addition, these methods are typically designed to handle uniform color deviations [43], making them to struggle with more complex underwater scenes that have diverse color distortions. This limitation can lead to issues such as new color biases, loss of fine detail, and uneven brightness across the image [62]. CNN-based methods are also computationally intensive, requiring significant processing power for both training and inference [49].

#### GAN-based.

GAN-based methods are widely used for image generation and enhancement, but their application to underwater image processing comes with several challenges. While GANs can generate synthetic training data, the generated images may not fully capture the complexities of real underwater conditions, leading to discrepancies in the enhancement process. Training of GANs is highly sensitive to the balance between the generator and discriminator networks, and the imbalances can result in mode collapse, where the generator produces a limited variety of images, or instability during training [73,74]. Although some methods use depth maps or statistical background color distributions to improve the realism of generated images [ 68,69], achieving complete and consistent enhancement remains a challenge. Furthermore, the use of unpaired training data, as seen in CycleGAN and its variants, can lead to artifacts and color distortions due to the inherently complexity of translating between underwater and in-air domains [70,71]. GAN-based methods are also computationally intensive, requiring substantial processing power and time for both training and inference. Additionally, GANs may struggle to generalize across diverse underwater scenes, resulting in reduced performance in novel conditions.

#### Transformer-based.

Transformer-based methods, which excel at capturing global interactions through self-attention mechanisms, offer a promising approach to underwater image enhancement. However, these methods often struggle with capturing fine-grained local details [75,76], which are crucial for high-quality image enhancement. While the integration of R-FNNs addresses this limitation to some extent, it remains a challenge [76]. These models are also computationally intensive, requiring significant memory and processing power. Additionally, these models need large amount of diverse training data, including different water depths, water clarity, and lighting. Without sufficient diverse training data, these methods tend to deliver inconsistent results.

#### DQL-based.

DQL-based methods, based on reinforcement learning, also face challenges. They require paired training data, which is often scarce and costly to obtain for diverse underwater environments. This data limitation reduces their ability to generalize, leading to poor performance in novel or unseen underwater conditions. In addition, DQL-based methods are computational demanding, requiring substantial hardware resources to train deep neural networks, which can pose challenges for real-time application and resource-constrained applications. Another key challenge is the design of effective reward functions and action spaces [77]. These must balance the need for objective image quality improvements with subjective aspects, such as natural color adjustments, which can vary depending on the scene. This makes it difficult to ensure consistent results across different underwater environments with varying lighting, turbidity, and depth.

Table 3 provides a summary of the shortcomings identified for each color correction methods used for enhancing underwater images. These challenges can be categorized into four main areas:

**Table 3:** Challenges faced by the color correction methods used for enhancing underwater images.

Underwater Image Color Correction Method	Data Dependency	Algorithmic Limitations	Computational Complexity	Performance Variability
Underwater optical imaging properties-based		✓	✓	✓
Color constancy-based		✓		✓
Red channel compensation-based		✓		✓
Adaptive color correction-based		✓		✓
Histogram equalization-based		✓		✓
Color transfer-based	✓		✓	✓
Multi-color spaces-based		✓		✓
Color opponent compensation-based		✓		✓
Fusion-based			✓	✓
CNN-based	✓		✓	✓
GAN-based	✓		✓	✓
Transformer-based	✓		✓	✓
DQL-based	✓		✓	✓

- **Data Dependency:** One of the most significant challenges in underwater image enhancement methods is their dependence on large amount of labeled training data. Obtaining such data is costly and time-consuming given the variability in underwater environments such as water clarity, depth, and lighting conditions. Additionally, the limited availability of paired training data, which is crucial for supervised learning approaches, restricts the development of robust models. This data constraint hinders the ability of many methods, particularly those based on deep learning, such as CNNs, GANs, and DQL, to generalize well to new and unseen underwater conditions. As a result, given the limited datasets for underwater images, these models tend to underperform in novel environments, limiting their practical applicability.
- **Algorithmic Limitations:** Many underwater image enhancement methods face several algorithmic limitations. They struggle to model the complex optical properties of underwater environments, such as light absorption and scattering. Some methods simplify color correction with unrealistic assumptions about light attenuation, as seen in red channel compensation-based methods. Color constancy-based methods overlook the non-uniform lighting underwater. Color transfer-based methods depend heavily on specific reference images, which can result in unrealistic color mapping when the reference and target images differ significantly in color distributions or lighting conditions. Multi-color spaces-based methods that leverage information from multiple color spaces introduce additional complexities. These algorithmic challenges limit the overall performance of underwater image enhancement methods.
- **Computational Complexity:** Many underwater image enhancement methods are computationally intensive, requiring significant processing power and time to achieve desired results. This computational complexity poses a major limitation for real-time applications, such as autonomous underwater vehicles (AUVs) or remote-operated vehicles (ROVs), where low-latency processing is essential. Methods based on deep learning, such as CNNs, GANs, and transformers, and multi-input fusion approaches are particularly resource-intensive, often requiring high-end graphic processing units (GPUs) and large amount of memory for both training and inference. Even more traditional techniques, such as underwater optical imaging properties, color transfer, and fusion-based approaches, can struggle with computational efficiency, especially when processing high-resolution images or large datasets. Therefore, designing a lightweight structure for these methods is critical for real-time application.
- **Performance Variability:** Performance variability issues are significant challenges in the field of underwater image enhancement. Almost all methods struggle to maintain consistent performance across varying underwater conditions, such as differences in water clarity, depth, and lighting. For example, a method trained in clear water conditions may not generalize well to low-light environments. Enhancement methods like CNN-based and GAN-based approaches may perform well on specific datasets but fail when applied to images from new or unseen conditions, leading to new color biases, artifacts, or detail loss. Methods such as adaptive color correction and red channel compensation also exhibit poor performance in environments where their core assumptions do not hold, further exacerbating the issues of inconsistent image quality. This variability limits the reliability and applicability of current enhancement methods in operational scenarios.

## Study taxonomy

**Fig 4:**
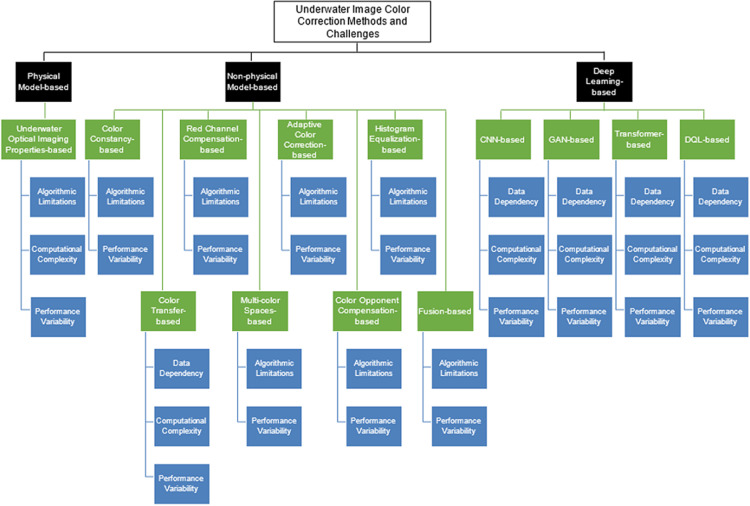
Taxonomy of underwater image color correction methods and challenges.

Fig 4 presents a taxonomy of color correction methods used in underwater image enhancement, based on the findings from the research questions. This taxonomy categorizes the various approaches aimed at addressing color deviation in underwater images, offering a structured overview that benefits the researchers, practitioners, and developers. By systematically classifying these methods, it highlights their core strategies and challenges, providing valuable insights into the field.

The taxonomy organizes the identified methods into three main categories, each addressing color deviation issues in underwater images with a different approach. The first category, physical model-based method, encompasses the underwater optical imaging properties-based. These methods attempt to correct color deviations by modeling the interactions of light and water, but they often face challenges related to algorithmic limitations, computational complexity, and performance variability across different underwater environments.

The second category, non-physical model-based methods, encompasses a range of approaches including red-channel compensation, color constancy, adaptive color correction, histogram equalization, color transfer, multi-color spaces, color opponent compensation, and fusion-based methods. Most of these methods share common limitations related to algorithmic limitations and performance variability.

Lastly, the third category is the deep learning-based methods, which includes CNN, GAN, transformer, and DQL-based. These methods leverage the deep learning approach to tackle the color correction problem, but their shortcomings include data dependency, computational complexity, and performance variability.

This taxonomy provides a structured framework for understanding various underwater image enhancement methods for color correction. By organizing these methods into physical model-based, non-physical model-based, and deep learning-based categories, it highlights the strengths and weaknesses of each category. Although progress has been made in this field, challenges such as data dependency, algorithmic limitations, computational complexity, and performance variability remain significant. This framework offers researchers and practitioners a useful guide to the current state of underwater image enhancement and areas for future development.

## Discussion

Various methods of underwater image color correction have been reviewed in this work. The methods can be grouped into three main categories: physical model-based, non-physical model-based, and deep learning-based approaches. This section elaborates on the differences in their strategies and discusses the challenges faced in correcting underwater color deviations. Physical model-based methods treat underwater image enhancement as an inverse imaging problem. They aim to reconstruct the original scene by compensating for the effects of light attenuation and scattering in water. These methods typically estimate factors like depth, transmission, and background light to correct color deviations [10–17]. Their effectiveness hinges on the accuracy of the physical models employed, which attempt to quantify the degradation mechanisms in underwater environments. Despite their solid theoretical foundation, physical model-based methods face several challenges:

- Developing models that accurately capture the complex interactions of light in water is inherently difficult, particularly under variable water conditions.
- These methods are often computationally intensive due to the need of detailed calculations related to light propagation.
- While these methods may perform well in controlled environments where water properties are well-known, they tend to struggle in more unpredictable or highly turbid conditions, leading to inconsistent results. Their performance is often scenario-specific, limiting their broader applicability.

Non-physical model-based methods handle underwater image degradation by directly manipulating pixel values, without attempting to model the physical processes that cause image degradation. These methods include a variety of techniques: color constancy, which adjusts color channels based on assumptions about the scene’s illumination [18–24]; red channel compensation, focused on recovering lost red channel information [20,25–27]; histogram equalization, which enhances contrast by redistributing pixel intensity values [39–47]; color transfer, which applies color statistics from a reference image to the underwater image [48–51]; multi-color space method, which extracts feature from multiple color spaces [46,52–55]; color opponent compensation, which adjusts color channels based on human color perception models [56,57]; and the fusion method, which combines multiple corrected versions of a single image to generate an enhanced result [18,20,22,25,29,38,45,50,55,58]. Non-physical model-based methods are simpler and often more efficient than physical model-based methods. However, they come with limitations:

- They rely on simplified assumptions, such as uniform lighting or the use of a specific reference image, such as uniform lighting or specific reference images, which can lead to inaccurate or incomplete corrections.
- Integrating multiple color spaces adds complexity, often resulting in inconsistent enhancement.
- While these methods can perform well in specific scenarios, they may struggle in extreme conditions such as uneven lighting and highly turbid water. This variability can lead to both under- and over-enhancement, affecting the consistency and quality of color correction across different underwater environments.

In recent years, deep learning-based methods have emerged as a promising trend for underwater image enhancement. These methods, including CNNs [43,46,49,52,53,59–66], GANs [57,67–74], transformers [75,76], and DQL [77], leveraging their ability to model complex, non-linear relationships in data. By learning from large datasets, these models can map degraded underwater images to their enhanced counterparts. Despite their potential, deep learning-based methods face significant challenges:

- These methods require extensive amount of labeled training data to perform effectively. The scarcity of real-world underwater datasets, combined with the reliance on synthesized data, limits their ability to generalize across different environments with varying levels of light attenuation and water clarity.
- Training and developing these models are resource-intensive, requiring powerful GPUs and substantial memory. This can make them unsuitable for real-time applications or environments with limited computational resources.
- While these models can excel on the datasets they are trained on, they often struggle to generalize to new, unseen underwater conditions, leading to inconsistent color correction. Additionally, their reliance on training data can result in overfitting to specific conditions, reducing their effectiveness in practical applications.

Each of the three method categories tackles color deviations in underwater images in distinct ways, and their effectiveness depends largely on the specific approach used with different strengths and limitations. Physical model-based methods prioritize recreating the underwater light propagation environment, but they struggle with high computational demands and generalizing to different underwater scenes. Non-physical model-based methods are more straightforward without attempting to model the physical processes that cause image degradation, but their reliance on simplistic assumptions may not always hold in all underwater environments. Deep learning-based methods presents a promising data-driven approach, yet they demanded substantial amount of training data and computational resources, limiting their feasibility for real-time or resource-constrained applications.

Evaluating the effectiveness of these methods is crucial for determining their success (IQA) is typically performed using both subjective and objective metrics. Subjective evaluation metrics reflect human visual perception and are also useful for comparing how well the enhancement meets human expectations. However, these methods are time-consuming and impractical for real-time applications.

Objective evaluation metrics have gained prominence due to their efficiency. They can be categorized into reference-based and non-reference-based metrics. While reference-based metrics require an ideal reference image, this is often unavailable in underwater scenarios. As a result, non-reference-based metrics, such as underwater image quality measure (UIQM) and underwater color image quality evaluation (UCIQE), are often preferred for underwater IQA. UIQM combines the measurements of underwater image colorfulness measurement (UICM), underwater image sharpness measurement (UISM), and underwater image contrast measurement (UIConM) [78]. UCIQE, on the other hand, assesses uneven color cast, noise, and blurring in underwater images [79]. Other common objective metrics include the structural similarity index measure (SSIM), patch-based contrast quality index (PCQI), mean square error (MSE), peak signal to noise ratio (PSNR), and entropy.

One of the main challenges in conducting this review includes the lack of standardization in the datasets and evaluation metrics across studies. This inconsistency complicates the comparison of results and hinders the establishment of universal conclusions. Additionally, for those methods involving multiple stages of processing, the lack of intermediate evaluations at each step limits the investigation of determining which stages contribute most to the overall correction.

This SLR includes studies published up to March 29, 2024, which may not reflect the most recent advances in underwater image enhancement. Given the rapid pace of development in this field, it is essential to acknowledge that the new technologies are constantly emerging.

Despite these limitations, this review offers a comprehensive overview of the current state of knowledge. It identifies ongoing challenges and areas that require further investigation. As the field evolves, more advanced techniques will be demanded to address existing limitations in underwater image color correction.

## Conclusion

This SLR identified 13 methods for underwater image color correction. These methods were categorized into three primary groups: physical models, non-physical models, and deep learning-based methods. Traditional approaches, like physical and non-physical model-based methods, have been foundational to the field, while deep learning-based methods have gained popularity in the recent five years.

Each method addresses color deviations in underwater images using different strategies. Physical model-based methods aim to reverse image degradation by simulating light attenuation and scattering in water. In contrast, non-physical model-based methods manipulate pixel values directly without considering these underlying physical processes. Deep learning-based methods use data-driven techniques to learn relationships between degraded and enhanced images from large datasets. Each category has its strengths, but challenges remain, such as algorithmic limitations, data dependency, computational complexity, and performance variability. Overcoming these challenges is critical for developing more robust and efficient solutions for underwater image enhancement.

This review provides a structured overview of the current state of knowledge in the field of underwater image enhancement for color correction. It highlights the strengths and limitations of existing methods and identifies gaps in the literature. Specifically, future studies should aim to address issues such as the lack of standardized datasets and evaluation metrics, improving the generalization of enhancement methods, and reducing computational requirements for real-time applications.

In conclusion, while significant progress has been made in underwater image enhancement, ongoing research and development are essential to overcoming the challenges of color deviations and improving the overall quality and applicability of these methods across different underwater environments.

## Supporting information

S1 Table PRISMA 2020 checklist. (DOCX)

S2 Table Assessment of study quality – MMAT tool. (DOCX)

S3 Table All studies identified. (XLSX)

S4 Table Included studies characteristics. (XLSX)
