# Supplementary material for: Color correction methods for underwater image enhancement: A systematic literature review
Source: PLoS One. 2025 Mar 10;20(3):e0317306. doi: 10.1371/journal.pone.0317306 (PMC11892867; doi:10.1371/journal.pone.0317306)
Supplement: S2 Table — (DOCX) [file pone.0317306.s002.docx]

**S2A Table**. Assessment of Study Quality – MMAT Tool

| **Quantitative Studies (MMAT Tool)** | **1.1: Is the sampling strategy relevant to address the research question?** | **1.2: Is the sample representative of the target population?** | **1.3: Are the measurements appropriate?** | **1.4: Is the risk of nonresponse bias low?** | **1.5: Is the statistical analysis appropriate to answer the research question?** | **Comments** |
| --- | --- | --- | --- | --- | --- | --- |
| Li [12] | Yes | Yes | Yes | Yes | Yes | - |
| Zhou [13] | Yes | No | Yes | Yes | Yes | The article does not discuss the details of datasets used. |
| Hou [14] | Yes | No | Yes | Yes | Yes | The article does not discuss the details of datasets used. |
| Zhou [15] | Yes | Yes | Yes | Yes | Yes | - |
| Nomura [17] | Yes | Yes | Yes | Yes | Yes | - |
| Ding [19] | Yes | No | Yes | Yes | Yes | The article does not mention the source of datasets used. |
| Ancuti [20] | Yes | No | Yes | Yes | Yes | The article does not mention the source of datasets used. |
| Lee [21] | Yes | No | Yes | Yes | Yes | The article does not mention the source of datasets used. |
| Qiang [22] | Yes | Yes | Yes | Yes | Yes | - |
| Yan [24] | Yes | Yes | Yes | Yes | Yes | - |
| Zhang [25] | Yes | Yes | Yes | Yes | Yes | - |
| Li [26] | Yes | No | Yes | Yes | Yes | The article does not mention the source of datasets used. |
| Wang [27] | Yes | Yes | Yes | Yes | Yes | - |
| Zhang [28] | Yes | Yes | Yes | Yes | Yes | - |
| Zhang [29] | Yes | Yes | Yes | Yes | Yes | - |
| Zhou [30] | Yes | No | Yes | Yes | Yes | The article does not discuss the details of datasets used. |
| Zhang [31] | Yes | Yes | Yes | Yes | Yes | - |
| Luo [32] | Yes | Yes | Yes | Yes | Yes | - |
| Zhang [33] | Yes | Yes | Yes | Yes | Yes | - |
| Lin [34] | Yes | Yes | Yes | Yes | Yes | - |
| Hu [35] | Yes | Yes | Yes | Yes | Yes | - |
| Zhang [36] | Yes | Yes | Yes | Yes | Yes | - |
| Liang [37] | Yes | Yes | Yes | Yes | Yes | - |
| Kanagavel [38] | Yes | No | Yes | Yes | Yes | The article does not discuss the details of datasets used. |
| Wang [40] | Yes | No | Yes | Yes | Yes | The article does not mention the source of datasets used. |
| Abdul Ghani [41] | Yes | No | Yes | Yes | Yes | The article does not mention the source of datasets used. |
| Zhang [42] | Yes | Yes | Yes | Yes | Yes | - |
| Fu [43] | Yes | Yes | Yes | Yes | Yes | - |
| Abdul Ghani [44] | Yes | No | Yes | Yes | Yes | The article does not mention the source of datasets used. |
| Bai [45] | Yes | Yes | Yes | Yes | Yes | - |
| Liu [46] | Yes | Yes | Yes | Yes | Yes | - |
| Gowda [47] | Yes | No | Yes | Yes | Yes | The article does not mention the source of datasets used. |
| Liang [48] | Yes | No | Yes | Yes | Yes | The article does not discuss the details of datasets used. |
| Yang [49] | Yes | Yes | Yes | Yes | Yes | - |
| Li [51] | Yes | Yes | Yes | Yes | Yes | - |
| Wang [52] | Yes | Yes | Yes | Yes | Yes | - |
| Lin [53] | Yes | Yes | Yes | Yes | Yes | - |
| Dong [54] | Yes | Yes | Yes | Yes | Yes | - |
| Zhou [55] | Yes | Yes | Yes | Yes | Yes | - |
| Ancuti [56] | Yes | Yes | Yes | Yes | Yes | - |
| Liu [57] | Yes | Yes | Yes | Yes | Yes | - |
| Kanagavel [58] | Yes | Yes | Yes | Yes | Yes | - |
| Wu [59] | Yes | Yes | Yes | Yes | Yes | - |
| Ji [60] | Yes | Yes | Yes | Yes | Yes | - |
| Liu [61] | Yes | Yes | Yes | Yes | Yes | - |
| Huang [62] | Yes | Yes | Yes | Yes | Yes | - |
| Zhou [63] | Yes | Yes | Yes | Yes | Yes | - |
| Huang [64] | Yes | Yes | Yes | Yes | Yes | - |
| Wang [65] | Yes | Yes | Yes | Yes | Yes | - |
| Lin [66] | Yes | Yes | Yes | Yes | Yes | - |
| Li [67] | Yes | Yes | Yes | Yes | Yes | - |
| Wu [68] | Yes | Yes | Yes | Yes | Yes | - |
| Cai [69] | Yes | No | Yes | Yes | Yes | The article does not discuss the details of datasets used. |
| Zhai [71] | Yes | No | Yes | Yes | Yes | The article does not mention the source of datasets used. |
| Yang [73] | Yes | Yes | Yes | Yes | Yes | - |
| Ma [74] | Yes | No | Yes | Yes | Yes | The article does not discuss the details of datasets used. |
| Peng [76] | Yes | Yes | Yes | Yes | Yes | - |

**S2B Table**. Assessment of Study Quality – CASP Tool

| **Qualitative Studies**  **(CASP Tool)** | **2.1: Was there a clear statement of the aims of the research?** | **2.2: Was the research design appropriate to address the aims of the research?** | **2.3: Was the data collected in a way that addressed the research issue?** | **2.4: Was the data analysis sufficiently rigorous?** | **2.5: Is there a clear statement of findings?** | **Comments** |
| --- | --- | --- | --- | --- | --- | --- |
| Liu [10] | Yes | Yes | No | No | Yes | The article does not provide information on data analysis, and it focuses more on conceptual proposal as well as theoretical analysis. |
| Chiang [11] | Yes | Yes | No | Yes | Yes | The article lacks a detailed description of data analysis process and does not demonstrate how the data support the findings. |
| Nomura [16] | Yes | Yes | No | No | Yes | The article does not provide information on data analysis, and it focuses more on conceptual proposal as well as theoretical analysis. |
| Ancuti [18] | Yes | Yes | Yes | No | Yes | There are less specific on how the analysis was conducted or how the findings from the experiments were synthesized. |
| Fu [23] | Yes | Yes | No | No | Yes | The article does not provide information on data analysis, and it focuses more on conceptual proposal as well as theoretical analysis. |
| Iqbal [39] | Yes | Yes | No | No | Yes | The article does not provide information on data analysis, and it focuses more on conceptual proposal as well as theoretical analysis. |
| Ancuti [50] | Yes | Yes | Yes | No | Yes | There are less specific on how the analysis was conducted or how the findings from the experiments were synthesized, and it focuses more on conceptual proposal as well as theoretical analysis. |
| Li [70] | Yes | Yes | Yes | Yes | Yes | - |
| Zhang [72] | Yes | Yes | Yes | Yes | Yes | - |
| Sun [77] | Yes | Yes | No | No | Yes | The article does not provide information on data analysis, and it focuses more on conceptual proposal as well as theoretical analysis. |
